# Chitosan as a Bio-Based Ligand for the Production of Hydrogenation Catalysts

**DOI:** 10.3390/molecules29092083

**Published:** 2024-05-01

**Authors:** Stefano Paganelli, Eleonora Brugnera, Alessandro Di Michele, Manuela Facchin, Valentina Beghetto

**Affiliations:** 1 Department of Molecular Sciences and Nanosystems, University Ca’ Foscari of Venice, Via Torino 155, 30172 Mestre, Italy; brugneraeleonora@gmail.com (E.B.); manuela.facchin@unive.it (M.F.); 2 Consorzio Interuniversitario per le Reattività Chimiche e la Catalisi (CIRCC), Via C. Ulpiani 27, 70126 Bari, Italy; 3 Dipartimento Fisica e Geologia, Università degli Studi di Perugia, Via Pascoli, 06123 Perugia, Italy; alessandro.dimichele@unipg.it; 4 Crossing S.R.L., Viale della Repubblica 193/b, 31100 Treviso, Italy

**Keywords:** chitosan, metal nanoparticles, catalysis, recyclable nanoparticles, platform chemicals

## Abstract

Bio-based polymers are attracting increasing interest as alternatives to harmful and environmentally concerning non-biodegradable fossil-based products. In particular, bio-based polymers may be employed as ligands for the preparation of metal nanoparticles (M(0)NPs). In this study, chitosan (CS) was used for the stabilization of Ru(0) and Rh(0) metal nanoparticles (MNPs), prepared by simply mixing RhCl_3_ × 3H_2_O or RuCl_3_ with an aqueous solution of CS, followed by NaBH_4_ reduction. The formation of M(0)NPs-CS was confirmed by Fourier Transform Infrared Spectroscopy (FT-IR), Differential Scanning Calorimetry (DSC), Thermal Gravimetric Analysis (TGA), Scanning Electron Microscopy (SEM), Energy-Dispersive X-ray Analysis (EDX), Transmission Electron Microscopy (TEM) and X-ray Diffraction (XRD). Their size was estimated to be below 40 nm for Rh(0)-CS and 10nm for Ru(0)-CS by SEM analysis. M(0)NPs-CS were employed for the hydrogenation of (*E*)-cinnamic aldehyde and levulinic acid. Easy recovery by liquid-liquid extraction made it possible to separate the catalyst from the reaction products. Recycling experiments demonstrated that M(0)NPs-CS were highly efficient up to four times in the best hydrogenation conditions. The data found in this study show that CS is an excellent ligand for the stabilization of Rh(0) and Ru(0) nanoparticles, allowing the production of some of the most efficient, selective and recyclable hydrogenation catalysts known in the literature.

## 1. Introduction

The environmental impact of fossil-based non-biodegradable materials is pushing for their substitution with bio-based ones [1,2,3,4,5,6]. Nevertheless, most products commercialized today are still produced from virgin naphtha [7,8], and their inadequate disposal has turned into a major environmental emergency [9,10]. The use of bio-based polymers appears to be a very interesting and eco-friendly alternative to the use of fossil-based polymers [11,12,13,14,15], and many examples are reported in the literature regarding the use of renewable and very abundant biopolymers, such as carboxymethyl cellulose, starch and chitosan, for different industrial applications [16,17,18,19,20,21,22,23]. In particular, chitosan (CS), is the second most abundant natural polymer, available in large quantities from food-industry waste, and is a renewable, environmentally friendly, biodegradable polymer. CS, mostly produced by deacetylation of chitin derived from the shells of shrimps and other crustaceans [24,25], is widely used by the food, medical, pharmaceutical and agricultural industries and can also be used for water treatment [26,27,28,29,30]. Its wide range of applications makes CS an appealing material, such that the global market for chitin and derivates is expected to reach US$63 billion by 2024 with production of about 1012–1014 tons/year [31,32].

Due to its a large number of aminic groups and its solubility in water, CS is also a very interesting biopolymer for the stabilization of transition metals for catalytic reactions. In fact, CS has been widely studied as a ligand for the preparation of homogeneous and heterogeneous catalysts employed for carbon-carbon coupling, oxidation, hydrogenation and click reactions, among others [25,33,34,35,36,37,38,39]. Concerning hydrogenation reactions, various examples have been reported in the literature regarding the use of chitosan as a ligand in the presence of Pd(0) and Pd(0) nanoparticles supported on silica [33,36,40,41,42], while Rh(0) and Ru(0) have been investigated less [43,44,45,46].

**Scheme 1:**
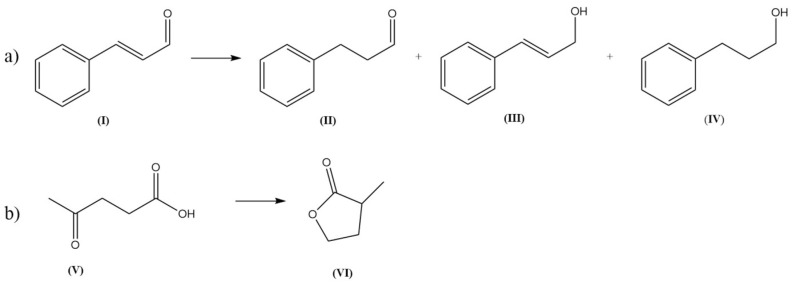
Hydrogenation reactions of (**a**) (*E*)-cinnamaldehyde (I) and (**b**) levulinic acid (V).

Based on our interest in the synthesis of fine chemicals by catalytic reactions, we deemed it interesting to study the efficiency of Rh(0) and Ru(0) chitosan nanoparticles (Rh(0)-CS, Ru(0)-CS) for the hydrogenation of (*E*)-cinnamaldehyde (**I**) and levulinic acid (**V**) (Scheme 1) [47,48,49,50]. In fact, (**I**) contains a C=C double bond and a C=O double bond, making it possible to evaluate both the activity and the selectivity of metal nanoparticles. In fact, hydrogenation of (**I**) may be employed either for the production of hydrocinnamaldehyde (**II**) or cinnamyl alcohol (**III**), which are both very interesting products with wide industrial applications [51,52,53,54]. Additionally, catalytic hydrogenation of levulinic acid makes it possible to produce γ-valerolactone (**VI**), a promising building block for fuel additives, resins, herbicides, pharmaceuticals, aromatic substances and chemical intermediates with wide application potential [47,48,49,50,51,52,53,54]. Therefore, (*E*)-cinnamaldehyde (**I**) and levulinic acid (**V**) are commonly employed as standard substrates to study the efficiency of hydrogenation catalysts. It is interesting to note that, although many studies have been reported in the literature employing both noble and non-noble homogeneous and heterogeneous catalysts for the hydrogenation of (**I**) and (**V**) [55,56,57,58], to the best of our knowledge, chitosan nanoparticles have never been employed for the hydrogenation of (**I**) or (**V**) [59,60,61,62,63].

With all this in mind, in this work, metal nanoparticles supported on chitosan (M(0)NPs-CS) were prepared by simply mixing RhCl_3_ × 3H_2_O or RuCl_3_ with an aqueous solution of CS, followed by NaBH_4_ reduction in analogy to the protocol previously reported by Harrad and co-workers for the preparation of Ni(0)-MNPs [64]. The formation of M(0)NPs-CS was verified by Fourier Transform Infrared Spectroscopy (FT-IR), Differential Scanning Calorimetry (DSC), Thermal Gravimetric Analysis (TGA), Scanning Electron Microscopy (SEM), Energy-Dispersive X-ray Analysis (EDX), Transmission Electron Microscopy (TEM) and X-ray Diffraction (XRD). Thus, the catalytic activity and selectivity of the Rh(0) and Ru(0)-CS nanoparticles was tested in water or water/organic medium for the hydrogenation of (I) and (V). Preliminary hydrogenation reactions in the presence of (I) were carried out to find the best operational conditions, to verify the selectivity of the MNPs towards C=C or C=O hydrogenation and to compare the efficiency of the MNPs studied in this work with data from the literature [65,66,67,68,69].

## 2. Materials and Methods

### 2.1. General Remarks

All commercially available reagents, solvents and chemicals were provided by Merck (Milan, Italy) and used as received. Chitosan (MW 90,000 Da), with a deacetylation degree of 0.90, was purchased from G.T.C. Bio Corporation (Qingdao, China). The catalysts and products were characterized by different analytical and spectroscopic analyses such as SEM, EDX, XRD, FT-IR, NMR and GC-MS. Scanning electron microscopy (SEM) was carried out using a FE-SEM LEO 1525 ZEISS (Jena, Germany). The acceleration potential voltage was maintained at 15 keV, and measurements were carried out using an in-lens detector. Samples were deposited on conductive carbon adhesive tape and metallized by sputtering with chromium (8 nm). Elemental composition and chemical mapping were determined using a Bruker Quantax EDX (Karlsruhe, Germany). TEM images were obtained using a Philips 208 transmission electron microscope (FEI, Hillsboro, OR, USA). The samples were prepared by putting one drop of an ethanol dispersion of the sample powder on a copper grid pre-coated with a Formvar film and dried in air. The average size distribution of droplets was determined with ImageJ software (LOCI, University of Wisconsin, Madison, WI, USA) using both SEM and TEM images.

FT-IR spectra were recorded on a Spectrum One (Perkin Elmer) in the 500–4000 cm^−1^ range. The samples were prepared using the KBr pellet method. TGA and DSC analysis were performed with a Linseis STA PT-1000 instrument (Messgeraete GmbH, Selb, Germany). XRD patterns were collected with a diffractometer in Bragg–Brentano geometry (Bruker D8 Advance, Bruker AXS GmbH, Karlsruhe, Germany), provided with a Lynxeye XE-T fast detector; CuKα radiation was used (operative conditions: 40 keV and 40 mA, step size 0.014° 2θ, step scan 10 s). Bruker DIFFRAC.EVA V5 software (Karlsruhe, Germay) equipped with the COD was used for the phase identification. The samples were prepared by placing the catalyst powder on a copper grid pre-coated with a Formvar film. The ^1^H and ^13^C NMR spectra of the products were registered on a Bruker UltraShield 400 spectrometer (Karlsruhe, Germany) operating at 400.0 and 101.0 MHz, respectively. The samples were prepared by dissolving reagents and products in deuterated chloroform (CDCl_3_). Gas-liquid chromatography analyses were performed on an Agilent 6850 gas chromatograph; gas chromatography–mass spectrometry analyses were performed on an HP 5890 series II gas chromatograph interfaced to a HP 5971 quadrupole mass detector. The samples were prepared by dissolving 3 drops of analyte in 2 mL of diethyl ether. ICP analyses were performed with the ICP MS OES DV 5300, Perkin Elmer (Milan, Italy).

### 2.2. Preparation of M(0)NPs-CS

The preparation of Rh(0) and Ru(0) nanoparticles stabilized with CS using NaBH_4_ as a reductant was carried out according to the same procedure for both metals, starting from RhCl_3_ × 3H_2_O and RuCl_3_ respectively. For example, the preparation of Rh(0)-CS (M/CS: 1/8 mol/mol) nanoparticles was as follows: 10 mL of distilled water and 367.0 mg (1.86 mmol) of chitosan hydrochloride were added into a 150 mL two-neck flask, equipped with a magnetic stirring bar, under nitrogen flow and left under stirring until a homogeneous solution was formed (about 1 h). Then, 61.0 mg (0.23 mmol) of RhCl_3_ × 3H_2_O and a solution of 200 mg (0.53 mmol) of NaBH_4_ dissolved in 50 mL of water were added under nitrogen into the reaction flask. The solution was left under stirring for 24 h and then centrifuged, and the solid was dried under vacuum overnight at RT. Ru(0)-CS was prepared analogously, using 47.7 mg (0.23 mmol) of RuCl_3_.

### 2.3. General Procedure for the Hydrogenation Reactions with M(0)NPs-CS

Hydrogenation experiments were carried out in a magnetically stirred stainless steel autoclave (total volume 150 mL) connected to a thermostatic bath in order to keep the reaction temperature constant within ± 1 °C. The same experimental protocol was used for hydrogenation reactions and recycling experiments, with all different substrates tested. For example, the procedure for the hydrogenation of (*E*)-cinnamaldehyde (**I**) was as follows (entry 1, Table 1). Under an inert atmosphere, in a 50 mL vial equipped with a small magnetic bar, we introduced 2 mL of water, 2 mL of THF, 104 mg (0.79 mmol) of (**I**), 10.3 mg (7.9 × 10^−3^ mmol) of catalyst and 12 mg (0.08 mmol) of undecane as an internal standard. Then, the vial was placed in a pre-purged 150 mL autoclave, and 10 atm of H_2_ was added. The autoclave was then heated at 80 °C and kept under constant magnetic stirring. After 16 h, the autoclave was cooled to room temperature and the residual gas vented off, and the reaction mixture was analysed by gas chromatography to calculate reaction conversion. Products were recovered from the reaction mixture by extraction with diethyl ether, then dried with anhydrous Na_2_SO_4_, followed by organic evaporation. Products were characterized by GC-MS, FT-IR and ^1^H and ^13^C NMR, and the data were compared to the literature [68,69,70,71]. The water solution was kept under nitrogen and used for recycling experiments. All experiments were performed in triplicate. All recycling experiments were carried out in the same reaction conditions as the first run, unless otherwise specified.

*Cinnamaldehyde* (**I**): ^1^H NMR (300 MHz, CDCl_3_): δ = 9.46 (d, 1 H), 7.30–7.36 (m, 2 H), 7.16–7.22 (m, 4 H), 6.48 (dd, 1 H); ^13^C NMR (101 MHz, CDCl_3_): δ = 193.8 (CH), 152.9 (CH), 134.1 (Cquat), 131.4 (CH), 129.2 (CH), 128.7 (CH), 128.6 (CH); GC-MS *m/z* 132.06 (M^+^, 100), 133.06 (M^+^, 9.9), 103 (M^+^ − CHO, 45), 77 (C_6_H_5_, 27).

*3-Phenylpropanal* (**II**): ^1^H NMR (CDCl_3_): δ = 9.74 (s, CHO, 1H), 7.24–7.10 (m, 5H), 2.91–2.86 (t, 2H), 2.72–2.67 (m, 2H); ^13^C NMR (101 MHz, CDCl3): δ = 201.5, 140.3, 128.6, 128.2, 126.3, 45.2, 28.1; GC-MS *m/z*: 134 [M]^+^; 105 [M − CHO]^+^; 91; 78 [M − C_3_H_4_O]^+^.

*Cinnamic alcohol* (**III**) ^1^H NMR (300 MHz, CDCl_3_): δ = 7.38–7.35 (m, 2H), 7.27–7.24 (m, 3H), 6.62–6.58 (m, 1H), 6.33–6.28 (m, 1H), 4.26–4.25 (m, 2H), 2.58 (s, 1H). ^13^C NMR (101 MHz, CDCl_3_): δ = 142.0, 128.6, 128.5, 126.0, 62.4, 34.4, 32.2; GC-MS *m/z* 134 [M]^+^; 116 [M − H_2_O]^+^; 91 [M − C2H3O]^+^; 78 [M − C3H4O]^+^;

*3-Phenylpropanol* (**IV**): ^1^H NMR (300 MHz, CDCl_3_): δ = 7.31–7.28 (m, 2H), 7.21–7.18 (m, 3H), 3.69–3.67 (t, 2H), 2.73–2.71 (m, 2H), 1.93–1.88 (m, 2H). ^13^C NMR (101 MHz, CDCl_3_): δ = 142.0, 128.6, 128.5, 126.0, 62.4, 34.4, 32.2; GC-MS *m/z* 136 [M]^+^; 118 [M − H_2_O]^+^; 105 [M − CH_2_OH]^+^; 91 [M − C_2_H5O]^+^; 77 [MC_3_H_7_O]^+^;

*Levulinic acid* (**V**): ^1^H NMR (300 MHz, CDCl_3_): δ = 11.01 (br s, 1H), 2.74 (t, *J* = 6.5 Hz, 2H), 2.62 (t, *J* = 6.5 Hz, 2H), 2.19 (s, 3H) ppm; ^13^C NMR (101 MHz, CDCl_3_): δ = 206.6, 178.7, 37.8, 29.9, 27.9 ppm; GC-MS *m/z* 116 [M]^+^; 87 [M − CHO]^+^; 73 [M − C_2_H_3_O]^+^;

*Γ-Valerolactone*: ^1^H NMR (300 MHz, CDCl_3_): δ = 82.06 (s.3H), 2.2 (m, 1H), 2.4 (m, 1H), 2.9 (m, 1H), 4.25 (m, 2H), 4.38 (m, 2H); ^13^C NMR (101 MHz, CDCl_3_): 821.1, 26.3, 39.8, 63.1, 67.4, 171.1, 177.1; GC-MS *m/z* 100 [M]^+^; 85 [M − CH_3_]^+^; 56 [M − C_2_H_4_O]^+^; 28 [C_2_H_4_]^+^.

## 3. Results and Discussion

### 3.1. Preparation and Characterization of M(0)NPs-CS

**Scheme 2:**
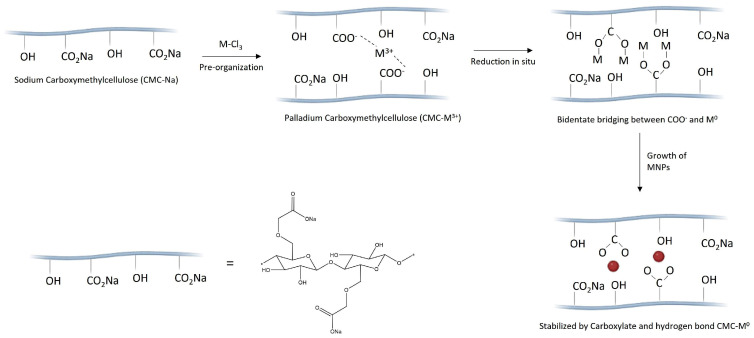
Proposed scheme of MNP formation by coordination of the metal to −COONa groups.

M(0)NPs-CS were easily prepared by adding the RhCl_3_ × 3H_2_O or RuCl_3_ catalyst precursor to an aqueous solution of CS at room temperature, followed by reduction with NaBH_4_ [64]. Pre-reduction with NaBH_4_ was carried out following a similar procedure to the one reported by Harrad and co-workers to prepare Ni(0) carboxymethylcellulose nanoparticles in a highly efficient manner [64,72]. Additionally, it may be supposed that formation of Rh(0) and Ru(0)-CS nanoparticles occurs in a similar manner to that reported by Xiao [73] for M(0)NPs and CMCNa (Scheme 2).

#### 3.1.1. Fourier Transform Infrared Spectroscopy (FT-IR) of MNP(0)-CS and Chitosan

**Figure 1:**
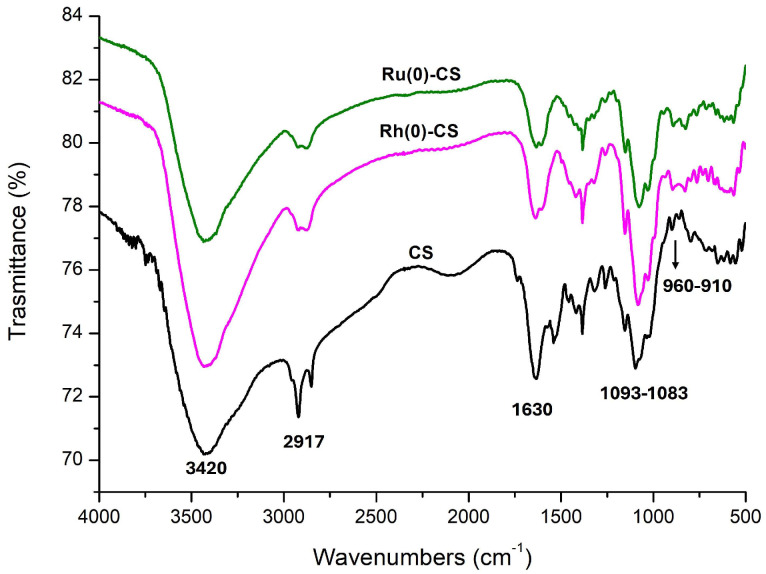
FTIR spectra of CS (black), Rh(0)-CS (purple) and Ru(0)-CS (green) in KBr.

Commercially available CS, Rh(0) and Ru(0)-CS nanoparticles were characterized by FT-IR (Figure 1). The intense band at 3420 cm^−1^ in the FT-IR spectrum of CS is characteristic of −OH functional group stretching, while stretching of the intramolecular hydrogen bonds of the polysaccharide and the axial −CH stretching are evidenced at 2917 cm^−1^. In this region, Rh(0)-CS and Ru(0)-CS nanoparticles show a similar FT-IR pattern, yet the signals are weaker and broader. This behaviour suggests a decrease in the intra-molecular hydrogen bonds between the chitosan chains, probably due to the presence of M(0)NPs. At 1630 cm^−1^, the typical absorption of the primary amide (C=O stretching) is identifiable, while adsorption bands between 1093–1083 cm^−1^ are attributed to the stretching of the polysaccharide skeleton. Signals of NH_2_ groups are present between 960–910 cm^−1^. Since only moderate differences were observed between CS and Rh(0) or Ru(0)-CS, further characterizations were carried out by DSC, TGA, SEM, EDX and XRD.

#### 3.1.2. Differential Scanning Calorimetry (DSC) and Thermal Gravimetric Analysis (TGA) of MNP(0)-CS and Chitosan

**Figure 2:**
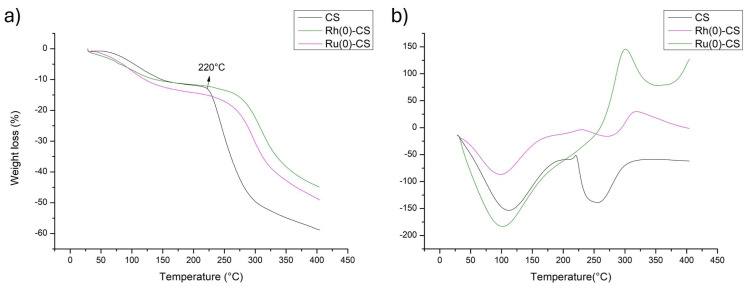
TGA (**a**) and DSC (**b**) profiles of CS (black), Rh(0)-CS (magenta) and Ru(0)-CS (green).

In agreement with the literature, TGA analysis of CS showed three characteristic temperature intervals with weight loss % between 10 and 50% (Figure 2). The first degradation occurs between 45 °C and 150 °C with 10–15% weight loss, corresponding to the evaporation of water physically adsorbed in CS. The second weight loss (about 50%) occurs between 220 °C and 300 °C, due to depolymerization or decomposition of polymer chains through deacetylation and cleavage of glycosidic linkages [74,75]. Finally, at temperatures above 300 °C, the residual carbon backbone and the chains of the polysaccharide collapse loosing further 10–15% weight [75,76] due to the pyrolytic degradation of chitosan, as well assessed in the literature [76]. In agreement with the literature, Rh(0)-CS and Ru(0)-CS showed lower decomposition rates, providing higher thermal stabilities than CS (Figure 2).

The DSC results of Rh(0)-CS and Ru(0)-CS were very similar to those of CS (Figures S1–S3), except for Ru(0)-CS, where the second endothermic peak occurred at higher temperatures than for CS (350 °C versus 250 °C), probably due to higher stability of the catalyst compared to CS and Rh(0)-CS.

#### 3.1.3. Scanning Electron Microscopy (SEM), Energy-Dispersive X-ray Analysis (EDX), Transmission Electron Microscopy (TEM) of MNP(0)-CS

**Figure 3:**
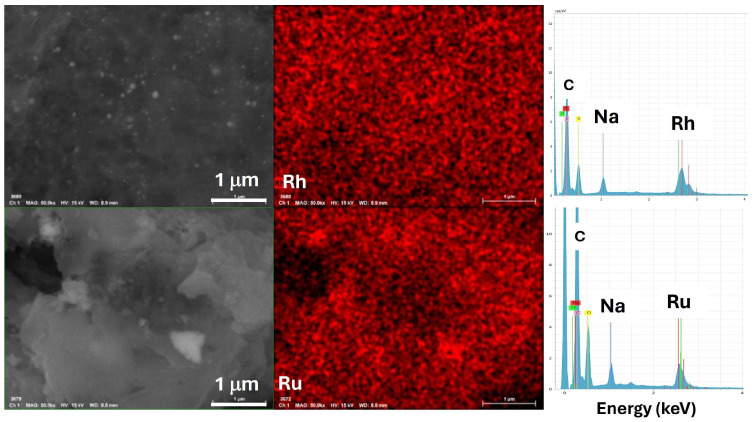
SEM (1 µm), EDX element mapping and energy spectrum of Rh(0)-CS (**top**) and Ru(0)-CS (**bottom**).

**Figure 4:**
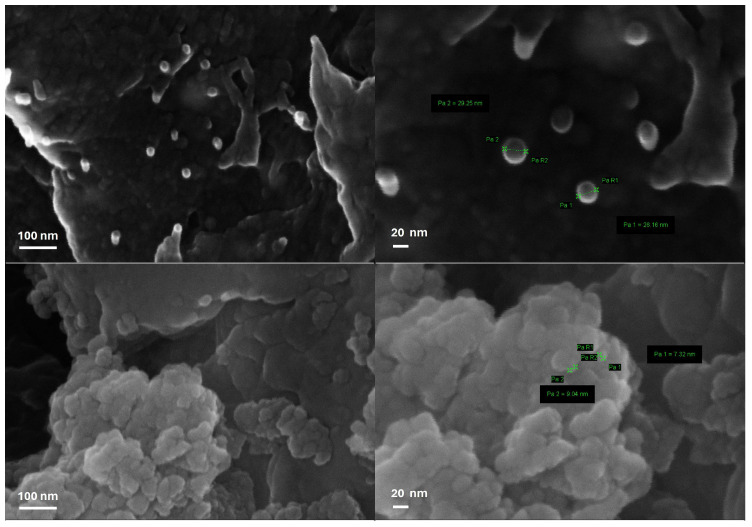
SEM images (100 nm and 20 nm) of Rh(0)-CS (**top**) and Ru(0)-CS (**bottom**).

Further characterizations were carried out by SEM and EDX. Mapping carried out on Rh(0)-CS and Ru(0)-CS by EDX (Figure 3 and Figure 4) evidenced a homogeneous dispersion of the metal within the polymeric matrix, and no regions with higher metal concentrations were observed, denoting the absence of nanoparticle aggregation.

SEM analysis further evidence that the metal particles have dimensions <40 nm in the case of Rh(0), suggesting the formation of aggregates, and <10 nm for Ru(0), confirming the homogeneous distribution within the polymer matrix (Figure 4).

**Figure 5:**
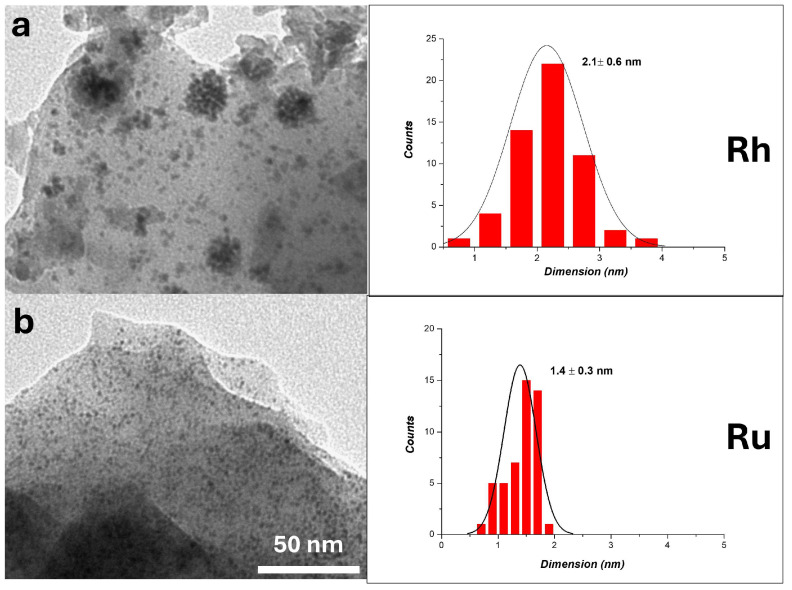
TEM images (50 nm) of Rh(0)-CS (**a**) and Ru(0)-CS (**b**).

In order to obtain a clear size evaluation of M(0)NPs (Figure 5), TEM images of the freshly prepared catalysts were acquired. Transmission electron microscopes are, in fact, capable of imaging at a significantly higher resolution than SEM devices and can be crucial for the size determination of M(0)-CS nanoparticles.

From TEM images on the fresh catalysts, the size of Rh(0)-CS was estimated to be around 2.1 nm, while Ru(0)-CS gave smaller nanoparticles of an average size of 1.4 nm. It was also evident that both Rh(0)-CS and Ru(0)-CS nanoparticles were homogeneously distributed in the chitosan, but the Rh(0)-CS nanoparticles tended to aggregate into 20 to 50 nm aggregates, confirming SEM results.

#### 3.1.4. X-ray Diffraction (XRD) of MNP(0)-CS

**Figure 6:**
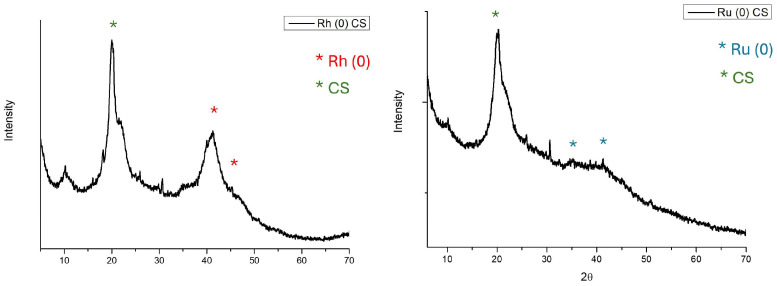
XRD patterns of Rh(0)-CS (**left**) and Ru(0)-CS (**right**).

X-ray diffraction (XRD) analysis results obtained for Rh(0)-CS and Ru(0)-CS are reported in Figure 5. Both graphs have in common the two characteristic peaks of chitosan (2θ = 10°, 19°), which indicate the high degree of crystallinity of the polymer. The absence of peaks below 2θ = 10° confirms the high deacetylation degree of the chitosan employed [77]. As regards Rh(0)-CS nanoparticles (Figure 6), typical peaks of nanometric-sized metallic Rh(0) are present at 2θ = 40°, 70° and 85° [78] (JCPDS card number 5-685).

In XRD of Ru(0)-CS, a peak at 2θ = 40°, characteristic of nanometric-sized metallic Ru(0), was present, while other relevant peaks were not evidenced [79].

### 3.2. Catalytic Hydrogenation in the Presence of M(0)NPs-CS

To study the activity of M(0)NPs-CS and optimize hydrogenation reaction conditions, preliminary experiments were carried out using (*E*)-cinnamaldehyde (**I**) as a model substrate and relevant data are reported in Table 1. As previously mentioned, the hydrogenation of (**I**) is challenging due to the possible formation of several by-products [44,80,81,82,83,84,85] and is often chosen as model reaction considering both its scientific and industrial relevance [84,85,86,87].

Preliminary hydrogenation reactions of (**I**) were performed in the presence of Rh(0)-CS with Rh/(**I**) mol/mol ratio = 1/100 at 80 °C and p(H_2_) = 10 atm for 16 h (entries 1–1r_3_, Table 1). Since (**I**) is not completely soluble in water, a solvent mixture 1/1 (vol/vol) of water/THF was used, the former to solubilize the catalyst and the latter as a solvent for the substrate and products.

Then, the influence of NaBH_4_ pre-reduction on Rh and Ru nanoparticles was investigated. A set of experiments were performed employing metal/CS solutions prepared in the absence of NaBH_4_, referred to as Rh(II)-CS and Ru(II)-CS, respectively. At 80 °C and p(H_2_) 10 atm, Rh(II)-CS gave decreased substrate conversions (<75%) and reduced selectivity in **II** (<50%) compared to nanoparticles pre-reduced with NaBH_4_ (see run 1, Table 1). A similar trend was observed employing Ru(II)-CS. Interestingly, recycling experiments showed that nanoparticles prepared without pre-reduction with NaBH_4_ totally lost catalytic activity, in contrast to pre-reduced M(0)NPs-CS. Although hydrogen employed during the catalytic reaction should reduce the metal centre with “in situ” generation of the M(0)NPs-CS, recycling experiments clearly show that this is not the case and that pre-reduction with NaBH_4_ is necessary to achieve highly active M(0)NPs-CS nano-catalysts. Further studies are ongoing to gain deeper understanding of this finding.

**Scheme 3:**
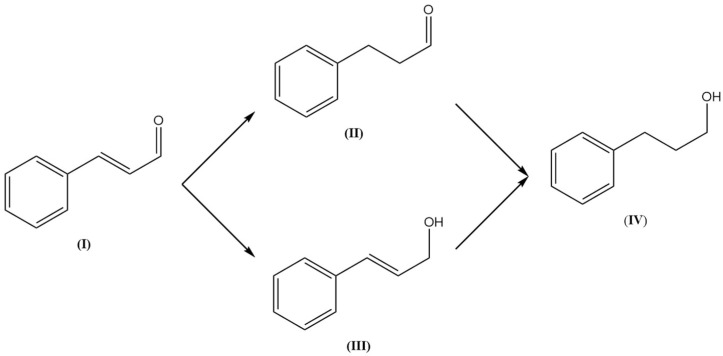
Schematic pathways of hydrogenation reaction of (*E*)-cinnamaldehyde (**I**).

The data reported in Table 1 show that, in the reaction condition tested, Rh(0)-CS makes it possible to achieve total conversion of **I** and high selectivity towards C=C hydrogenation to aldehyde (**II**) even after one recycling experiment, the second most abundant product being 3-phenylpropanol (**IV**). From the second recycling experiment, both activity and selectivity decreased (entries 1–1r_3_, Table 1). According to Scheme 3, **IV** may be formed by hydrogenation of II or III. The data reported in Table 1 show that in the presence of the Rh(0)-CS nanoparticles tested, the increase in **III** formed from runs 1 to 1r3 was predominantly at the expense of II and might be due to partial deactivation and loss of selectivity of recycled Rh(0)-CS nanoparticles.

Further recycling led to the formation of increasing quantities of cinnamyl alcohol (**III**), probably as a consequence of partial catalyst deactivation. Reducing reaction time (entry 2, Table 1), a drastic drop in conversion was recorded, a drop that was also observed by decreasing either hydrogen pressure (entry 3, Table 1) or temperature (entry 4, Table 1) or by reducing catalyst loading (catalyst/substrate molar ratio 1/200) (entry 5, Table 1). It should be noted that, despite the reduced activity of the catalyst in these reaction conditions, higher selectivity towards the carbon–carbon double bond hydrogenation product was nevertheless obtained, and in the best reaction conditions, total selectivity in (**II**) was achieved (entry 5, Table 1).

Hydrogenation of (**I**) with Ru(0)-CS, carried out in the same reaction conditions employed in the presence of Rh(0)-CS, highlighted the reduced activity of this catalytic species compared to analogous Rh(0)-CS, albeit with comparable selectivity (compare entries 1 and 6, Table 1). Similar results were obtained when increasing p(H_2_) pressure from 10 to 20 atm (compare entries 6 and 7, Table 1), while at higher temperatures (100 °C), higher conversion could be achieved at the expense of selectivity (entry 8, Table 1). When p(H_2_) was raised to 20 atm, conversion was almost complete (97%), with higher selectivity towards 3-phenylpropanol (**IV**) (57%). Interestingly, recycling of the catalyst lead to an increase in selectivity in the fully hydrogenated product (**IV**) from 57% in the first experiment to 82% after the third recycling (compare entries 9–9r3, Table 1). Further, the high conversion obtained for all rounds of recycling confirms the stability of the Ru(0)-CS catalytic species. In agreement with the literature, Ru(0)-CS nanoparticles were active in more extreme conditions than Rh(0)-CS nanoparticles but were more recyclable than the latter [44].

Since no data are available on the hydrogenation of (**I**) with CS nanoparticles, in order to evaluate the efficiency of Rh(0) and Ru(0)-CS as tested in this work, was comparison with heterogeneous Rh(0) and Ru(0) catalysts reported in the literature was carried out [44,88]. According to a very recent work by Patil and co-workers, results achieved with Rh(0) and Ru(0)-CS are particularly interesting, since, in most cases, heterogeneous catalysts reported in the literature have modest activity and selectivity, requiring harsh conditions and environmentally unfriendly organic solvents [44,86,87,88,89,90,91,92,93,94,95].

For example, Liu reported the use of Rh@MIL-101 (Cr), a metal–organic framework (MOF) with high surface area and porosity, for the hydrogenation of (**I**) [89]. At 30 °C, p(H_2_) 10 atm, with a Rh/(**I**) molar ratio of 1/400, in ethanol as a solvent, conversion rates of up to 98% of (**I**) were achieved by 5 h, with selectivity in (**II**) of 99%. In analogous reaction conditions, commercially available Rh/C gave significantly lower conversions (53%) and selectivity in (II) (89%) [44]. Although Rh@MIL-101 (Cr) can be recycled up to two times with almost no change in activity and selectivity, its sustainability is low considering that MIL is synthesized using toxic chemicals such as *N*,*N*-dimethylformamide (DMF) or hydrofluoric acid (HF) [94]. Alternatively, Rh(0) porphyrins have been used for the hydrogenation of (**I**), but with modest selectivity in (**II**) (≤80%). Additionally, toluene was used together with water as reaction solvent and NEt_3_ had to be added to promote the solubility in water of the Rh porphyrin complex [93].

To widen the scope of the reaction further hydrogenation tests were carried out in the presence of levulinic acid (**V**) (Scheme 1). Levulinic acid is commonly employed as standard substrate to verify the efficiency of new protocols intending to valorise bio-based feedstocks to produce sustainable, bio-based building blocks in alternative to fossil-based ones. It is important to note that in 2004 the US Department of Energy, ranked levulinic acid as one of the twelve most important platform chemicals derived from biomass [94,95].

For example, several studies have been reported in the literature for the use ruthenium supported on carbon [96], ZrO_2_ [63], TiO_2_ [65] or alumina [97] as catalysts for the synthesis of (**VI**) from levulinic acid.

Since (**V**) is soluble in water, hydrogenation reactions were carried out in water as solvent and at the end of the reaction the products were extracted from the water solution with diethyl ether, the catalyst remaining confined in the aqueous phase, ready to be reused.

The catalytic tests reported in Table 2 showed complete conversion for the substrate (**VI**) at 80 °C, p(H_2_) 20 atm for 16 h, using Rh(0)-CS with a Rh(0)/(**V**) ratio of 1/100 (entry 1, Table 2). Total conversion was also obtained at lower hydrogen pressure and temperature (see entries 2 and 3, Table 2). However, in these reaction conditions, a significant decrease in the Rh(0)-CS activity was registered upon recycling (entry 3r_1_, Table 2). In order to verify whether the loss of the Rh(0)-CS activity could be a consequence of catalyst leaching, ICP analysis of the organic solution was carried out, revealing negligible traces of metal (<0.1%). If hydrogen pressure was further decreased from 10 to 5 atm at 50 °C, conversion into (**VI**) was reduced (36%), although total selectivity was maintained (entry 5, Table 2).

Interestingly, experiments carried out in the same reaction conditions reported in entry 5 of Table 2, but in the presence of Ru(0)-CS, clearly demonstrate that Ru(0)-CS is highly active for the hydrogenation of (**V**), giving total selectivity towards the desired product (**VI**), such that it was possible not only to lower p(H_2_) to 1 atm but also to recycle the catalyst up to three times with no loss of activity or selectivity (see entries 7–9r_3_, Table 2). With the aim of minimizing the consumption of Ru(0)-CS, further experiments were performed with a Ru/(**V**) ratio of 1/200 mol/mol, at 50 °C, p(H_2_) 5 atm, for 4 h, yet in these reaction conditions, the activity of the Ru(0)-CS catalyst upon recycling decreased to 87% (see entries 10–10r_3_, Table 1).

Thus, further hydrogenation reactions were performed in more drastic conditions (entries 11–11r_3_, Table 2), but with an even lower Ru/(**V**) mol/mol ratio (1/500). These experiments showed that at 80 °C, p(H_2_) 20 atm by 16 h, even lower catalyst concentrations could be used, making it possible to achieve total substrate conversion and product selectivity even after three recycling tests.

As in the case of (*E)*-cinnamyl aldehyde (**I**) (see Table 1), the data achieved for the hydrogenation of levulinic acid (**V**) with Ru(0)-CS (see Table 2) are extremely interesting and reveal high performance compared to data in the literature [61,98,99]. In fact, Ndolomingo and co-workers recently reported a comparison between the efficiency values of different MNPs employed for the hydrogenation of (**V**) [91], and from this work it emerged that Ru@Meso-SiO_2_ nanoparticles are among most efficient hydrogenation catalysts, leading to total substrate conversion and selectivity in (**VI**) at moderate p(H_2_) (10 atm) and in a short time (5 h), yet very high temperatures are required (150 °C), and dioxane is used as a solvent [100]. Ru/SiO_2_, Ru/Al_2_O_3_, Ru/ZnO_2_ and Ru/TiO_2_ have been reported by Tan for the hydrogenation of (**V**), but in this case as well, high temperatures are required to achieve high product yields [67]. It should also be noted that in agreement with the literature, Rh(0)-CS nanoparticles gave the highest activity and selectivity for the synthesis of (**II**), i.e., towards C=C hydrogenation, in contrast to Ru(0)-CS nanoparticles, which gave the best results for the hydrogenation of C=O to give (**VI**).

## 4. Conclusions

In this work, water-soluble M(0)NPs-CS were prepared starting from RhCl_3_ × 3H_2_O or RuCl_3_ in an aqueous environment using chitosan (CS) as a ligand, pre-reduced before use with NaBH_4_. FT-IR of M(0)NPs-CS showed no substantial differences from CS, while TGA highlighted higher stabilization of Rh(0)-CS and Ru(0)-CS than CS. As regards EDX analysis, Rh(0)-CS and Ru(0)-CS appeared to be homogeneously dispersed within the polymer matrix, and no areas of higher metal concentration were noted, as would be observed in the case of nanoparticle aggregation. SEM and XRD analyses further confirmed that the particles had dimensions on the order of nanometres.

The catalytic efficiency of Rh(0)-CS and Ru(0)-CS nanoparticles was tested in hydrogenation reactions of model substrates such as (*E*)-cinnamaldehyde (**I**) and levulinic acid (**V**), important platform chemicals of strong industrial interest. Rh(0)-CS made it possible to achieve complete conversions of (**I**) at 80 °C and p(H_2_) 10 atm by 16 h, with selectivity in (**II**) of up to 84%, although the latter detectably decreased upon recycling. Additionally, for the hydrogenation of levulinic acid (**V**), complete conversions were obtained with Rh(0)-CS at lower reaction temperatures (50 °C).

In contrast, Ru(0)-CS was less active for the hydrogenation of (**I**), requiring higher reaction temperatures than Rh(0)-CS, yet leading to higher selectivity in (**III**) (91–100%). Interestingly, Ru(0)-CS was more efficient for the hydrogenation of levulinic acid (**V**), giving total conversion and selectivity in γ-valerolactone (**VI**) even after three recycling tests. Comparison with literature data highlighted that M(0)NPs-CS have very high performances compared to the best heterogeneous catalysis known for the hydrogenation of (*E*)-cinnamaldehyde and levulinic acid. Further studies are ongoing in the presence of other water-soluble bio-derived substrates, bearing suitable functional groups for the anchoring of M(0)NPs, in order to compare their catalytic efficiency to those of M(0)NPs-CS as reported in this work.

## Figures and Tables

**Table 1:** Hydrogenation of (**I**) in the presence of Rh(0) and Ru(0)-CS.

Entry	MNP(0)-CS	p(H_2_) (atm)	T (°C)	Conv. (%) ^a^	II(%) ^a^	III(%) ^a^	IV(%) ^a^
1	Rh(0)-CS	10	80	100	84	3	13
1r_1_				100	82	0	18
1r_2_				96	71	15	14
1r_3_				97	71	14	15
2 ^b^	Rh(0)-CS	10	80	32	91	3	6
3	Rh(0)-CS	5	80	52	81	13	6
4	Rh(0)-CS	10	50	25	96	0	4
5 ^c^	Rh(0)-CS	10	80	19	100	0	0
6	Ru(0)-CS	10	80	45	78	9	13
7	Ru(0)-CS	20	80	53	60	17	23
8	Ru(0)-CS	10	100	62	52	21	27
9	Ru(0)-CS	20	100	97	20	23	57
9r_1_				97	19	22	56
9r_2_				95	13	0	87
9r_3_				97	14	2	84
9r_4_				96	13	5	82

Reaction conditions: substrate (**I**): 25.4 mg (0.79 mmol); Rh/(**I**): 1/100 mol/mol; solvent: 2.0 mL H_2_O and 2.0 mL THF; t: 16 h, r: recycling experiment. ^a^ Data determined by GLC with undecane as internal standard. ^b^ t: 6 h. c Rh/(**I**): 1/200 (mol/mol).

**Table 2:** Hydrogenation of levulinic acid in the presence of M(0)NPs-CS.

Entry	MNP(0)-CS	M/S	t(h)	p(H_2_) (atm)	T (°C)	Conv (%) ^a^	VI (%) ^a^
1	Rh(0)-CS	1/100	16	20	80	100	100
2	Rh(0)-CS	1/100	16	10	80	100	100
3	Rh(0)-CS	1/100	16	10	50	100	100
3r_1_		1/100				68	68
4	Rh(0)-CS	1/100	16	5	50	36	36
5	Ru(0)-CS	1/100	16	5	50	100	100
6	Ru(0)-CS	1/100	4	5	50	100	100
7	Ru(0)-CS	1/100	4	2	50	100	100
7r_1_						100	100
8	Ru(0)-CS	1/100	4	2	50	100	100
9	Ru(0)-CS	1/100	4	1	50	100	100
9r_1_						99	99
9r_2_						99	99
9r_3_						99	99
10	Ru(0)-CS	1/200	4	5	50	100	100
10r_1_						87	87
10r_2_						85	85
10r_3_						85	85
11	Ru(0)-CS	1/500	16	20	80	100	100
11r_1_						100	100
11r_2_						100	100
11r_3_						100	100

Reaction conditions: substrate: 0.59 mmol; solvent: 4.0 mL H_2_O. ^a^ Data determined by GLC with undecane as internal standard. r: recycling experiment.

## Data Availability

The original contributions presented in the study are included in the article/Supplementary Material, further inquiries can be directed to the corresponding authors.

## Supplementary Materials

The following supporting information can be downloaded at: https://www.mdpi.com/article/10.3390/molecules29092083/s1, Figure S1: DSC and TGA profiles of Chitosan Hydrochloride; Figure S2: DSC and TGA profiles of Rh(0)-CS; Figure S3: DSC and TGA profiles of Ru(0)-CS.
