# Supplementary material for: Chitosan as a Bio-Based Ligand for the Production of Hydrogenation Catalysts
Source: Molecules. 2024 May 1;29(9):2083. doi: 10.3390/molecules29092083 (PMC11085195; doi:10.3390/molecules29092083)
Supplement: Supplementary file 1 [file molecules-29-02083-s001.zip › molecules-2960727-supplementary.pdf]

Chitosan as a Bio-based Ligand for the Production of Hydrogenation Catalysts

Stefano Paganelli[1,2]*, Eleonora Brugnera[1], Alessandro Di Michele[3], Manuela Facchin[1], and Valentina Beghetto[1,2,4]*

[1] Department of Molecular Sciences and Nanosystems, University Ca' Foscari of Venice, Via Torino 155, 30172 Mestre, Italy.

[2] Consorzio Interuniversitario per le Reattività Chimiche e la Catalisi (CIRCC), via C. Ulpiani 27, 70126 Bari, Italy.

[3] Università degli Studi di Perugia, Dipartimento Fisica e Geologia, Via Pascoli, 06123 Perugia, Italy.

[4] Crossing S.r.l., Viale della Repubblica 193/b, 31100 Treviso, Italy.

*Corresponding Authors: beghetto@unive.it; Tel.: +39-041-234-8928; spag@unive.it; Tel.: +390412348592

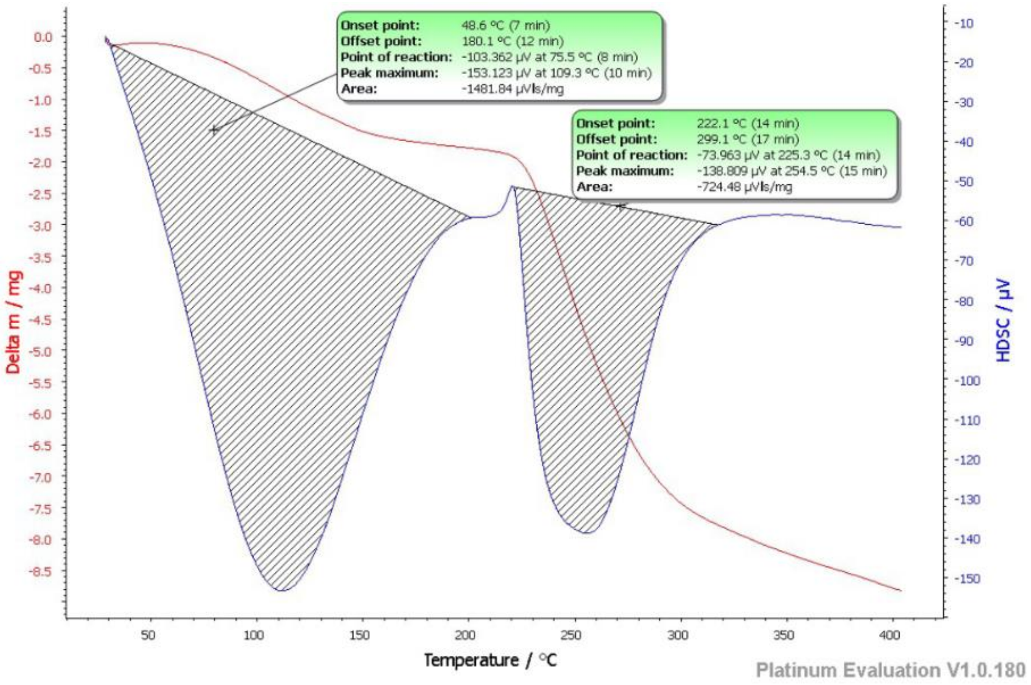

Figure S1 DSC and TGA profiles of Chitosan Hydrochloride

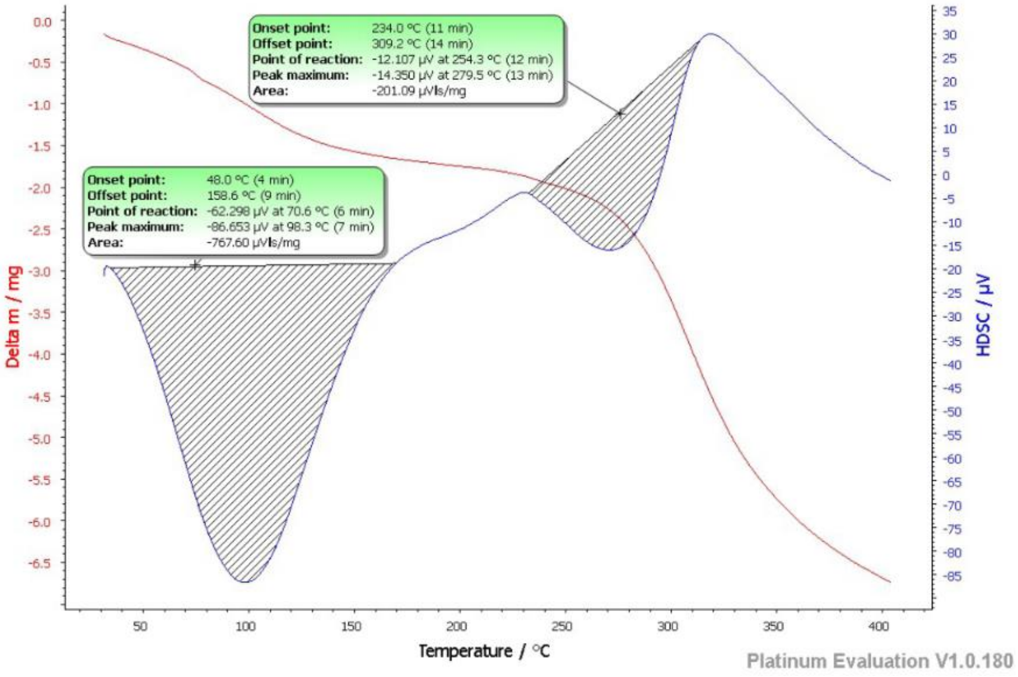

Figure S2 DSC and TGA profiles of Rh(0)-CS.

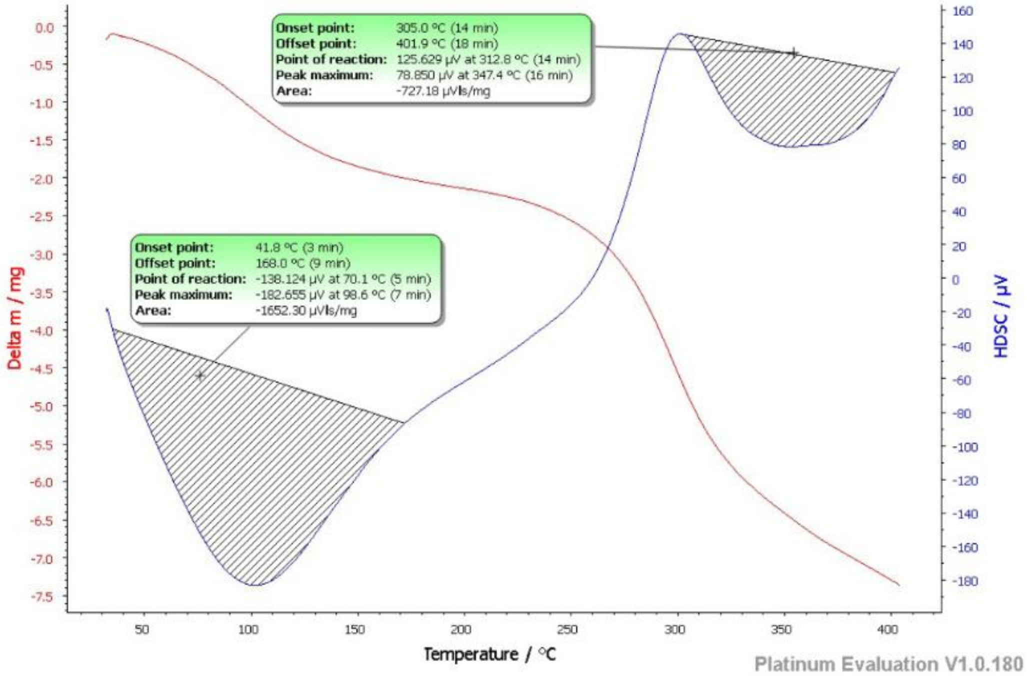

Figure S3 DSC and TGA profiles of Ru(0)-CS.
